# Highly Deep Ultraviolet–Transparent h-BN Film Deposited on an Al_0.7_Ga_0.3_N Template by Low-Temperature Radio-Frequency Sputtering

**DOI:** 10.3390/ma12244046

**Published:** 2019-12-05

**Authors:** Guo-Dong Hao, Manabu Taniguchi, Shin-ichiro Inoue

**Affiliations:** Advanced ICT Research Institute, National Institute of Information and Communications Technology (NICT), Kobe 651-2492, Japan; gd.hao@nict.go.jp (G.-D.H.); manabu.taniguchi@nict.go.jp (M.T.)

**Keywords:** hexagonal boron nitride, highly DUV–transparent material, RF sputtering, low-temperature deposition

## Abstract

Hexagonal boron nitride (h-BN) is an attractive wide-bandgap material for application to emitters and detectors operating in the deep ultraviolet (DUV) spectral region. The optical transmittance of h-BN in the DUV region is particularly important for these devices. We report on the deposition of thick h-BN films (>200 nm) on Al_0.7_Ga_0.3_N templates via radio-frequency sputtering, along with the realization of ultrahigh transmittance in the DUV region. The fraction of the gas mixture (Ar/N_2_) was varied to investigate its effects on the optical transmittance of BN. DUV light transmittance of as high as 94% was achieved at 265 nm. This value could be further enhanced to exceed 98% by a post-annealing treatment at 800 °C in a N_2_ ambient for 20 min. The phase of the highly DUV–transparent BN film was determined to be a purely hexagonal structure via Raman spectra measurements. More importantly, these deposition processes were performed at a low temperature (300 °C), which can provide protection from device performance degradation when applied to actual devices.

## 1. Introduction

In recent years, AlGaN-based ultraviolet and deep ultraviolet (DUV) light-emitting diodes (LEDs) have attracted considerable interest among the efforts to replace toxic mercury lamps in a broad range of applications, including water/air purification, sterilization, and medical devices [1,2,3]. One major obstacle to the realization of high efficiency in these devices is the lack of a transparent p-type contact layer [4,5,6], which means that the conventional techniques used to enhance the light extraction efficiency of GaInN-based visible LEDs are ineffective in DUV LEDs. Hexagonal boron nitride (h-BN) is an ultra-wide-bandgap (*E*_g_ ~6 eV) material in the group III nitride family [7,8]. More importantly, h-BN has a unique propensity for p-type doping. Previous reports have shown that good p-type conductivity can be achieved in h-BN via doping with Zn, Be, and Mg [9,10,11,12]. It has also been reported that the boron vacancies are acceptor-like defects with an energy level of approximately 150 meV above the valence band edge [13]. Strong p-type behavior has been demonstrated experimentally in undoped h-BN with a hole concentration of 10^20^ cm^−3^ at room temperature by the formation of boron vacancies grown by molecular beam epitaxy [14]. In addition, h-BN has an ideal band alignment to Al_0.7_Ga_0.3_N that allows it to act as a p-cladding layer for both hole injection and electron blocking [15]. These properties mean that h-BN is a promising candidate material for use as a p-type layer in optical devices. There have been a number of reports of h-BN layer synthesis using chemical vapor deposition (CVD) methods [16,17,18,19]. In these studies, the favored substrate materials are mostly metals such as Ni, Cu, or Fe, which are used as catalytic layers to promote h-BN film formation. However, the h-BN film thickness in these cases was limited to a few monolayers, which is insufficient to support carrier transport in device applications. Additionally, growth of h-BN layers on sapphire and silicon substrates has also been demonstrated [20,21,22]. Much of the recent interest in h-BN was stimulated by the report of growth of a thick h-BN film on an AlGaN layer by metal organic chemical vapor deposition (MOCVD) using a buffer layer [23]. This h-BN film is expected to be used as a p-type layer in DUV LED applications. However, the use of the buffer layer brings an extra high resistance that could cause extremely high device voltages. In addition, the MOCVD growth process required very high temperature conditions (>1300 °C), which can cause previously grown active regions to degrade and can also cause a chemical reaction at the AlGaN surface. These issues have limited the applicability of h-BN to optoelectronic devices and light output has thus not been realized from p-type h-BN devices to date. A new method to grow thick h-BN layers on AlGaN is therefore required. Sputtering is an alternative growth method that can deposit good-quality films at lower temperatures [24]. In this technique, high-energy ions are generated by a glow discharge in a low-pressure gas. The energetic ions eject source atoms from the targets and then deposit them on the substrate in a vacuum system. The source of energy is provided by energetic ions. Hence, sputter deposition can be used to deposit good-quality film at low temperatures, even down to room temperature. A radio frequency (RF) source is used because it can be used for not only conductive but also non-conductive targets [25]. One requirement that applies to h-BN in optical device applications is that it has high optical transmittance because the transmittance strongly determines the power conversion efficiency via the light extraction efficiency. While there have been several previous reports of sputter deposition of h-BN [9,26], the optical transmittance values of the reported layers in the DUV region were very low when compared with that of similar layers grown by MOCVD. In this paper, we report the realization of thick h-BN layers with high transmittance in the DUV region on an Al_0.7_Ga_0.3_N template that were deposited by RF magnetron sputtering under low temperature conditions. The transmittance of the 200-nm-thick h-BN film ultimately reaches as high as 98% at 265 nm. To the best of our knowledge, h-BN films prepared under low temperature conditions that demonstrate such high transmittance in the DUV region have not been reported to date.

## 2. Materials and Methods

We used commercial *c*-plane Al_0.7_Ga_0.3_N templates that were provided by LumiGNtech Co., Ltd. (Gwangmyeong-si, Korea) as the substrates [27]. We anticipated that the h-BN could be integrated with current AlGaN-based DUV optoelectronic devices and thus the specific template composition was selected to be identical to that of the p-type Al_0.7_Ga_0.3_N cladding layer that is typically used in 260–280 nm DUV LEDs [28]. All substrates were cleaned ultrasonically in acetone and then in isopropyl alcohol (IPA) followed by deionized water before being loaded into the chamber. The deposition of the h-BN films was performed using an RF (13.56 MHz) magnetron sputtering system (ULVAC MNS-2000). A radiation lamp that was located behind the substrate heated the substrate to the deposition temperature at a heating rate of 10 °C/min. The base pressure in the chamber was below 3 × 10^−5^ Pa. A high-purity sintered BN target (99.5% purity) with a diameter of 50 mm was used. The distance between the target and the substrates was 70 mm. The RF power applied to the target during deposition was maintained at 200 W. The sputtering was carried out in argon (Ar), nitrogen (N_2_), or a gas mixture of N_2_ and Ar. The N_2_/(N_2_ + Ar) fraction in the mixed gas was adjusted by varying the Ar and N_2_ flow rates, while the total gas flux was maintained at 30 SCCM at a pressure of 0.6 Pa in the chamber. The target was first pre-sputtered for 30 min for cleaning purposes with a shutter placed between the target and the substrate. During the pre-sputtering stage, the substrates were heated to 300 °C for BN deposition. The deposition duration was 30 min for all samples. After cooling to room temperature, some samples were subsequently transferred into a quartz tube for post-annealing treatment at 800 °C for 20 min in a flowing N_2_ ambient using a conventional rapid thermal annealing (RTA) method.

The deposition rate was calculated from the film thickness, which was measured using a scanning electron microscope (SEM). The optical transmittance of the h-BN films was characterized using a spectrophotometer operating in the 200–1000 nm range. The crystalline phase of the BN thin film was identified by Raman spectroscopy (using an inVia Raman Microscope, Renishaw, Gloucestershire, UK). The excitation source used in the Raman scattering measurements was a 532 nm laser.

## 3. Results and Discussion

**Figure 1:**
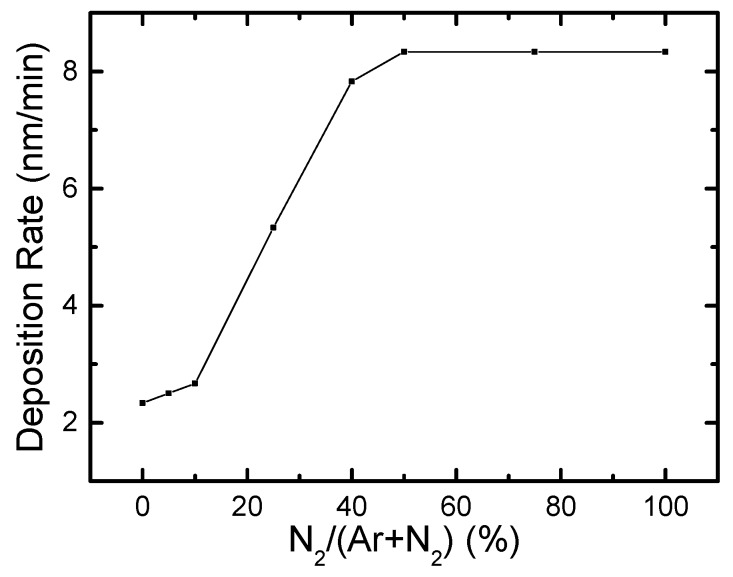
Deposition rate as function of N_2_/(N_2_ + Ar) gas flow ratio.

The dependence of the deposition rate on the N_2_ flow ratio during sputtering is illustrated in Figure 1. The results show that the deposition rate initially increases from 2.3 nm/min to 8.3 nm/min when the N_2_ flow ratio increases from 0 to 50%. Further increases in the N_2_ flow rate do not cause any further changes in the deposition rate. A similar trend was also reported in an earlier project for RF-sputtered boron carbonitride (BCN) films [29]. This behavior can be explained as being caused by nitridation of the BN target during these sputtering processes. Nitrogen vacancies can easily be generated and thus change the composition at the target surface during sputtering processes. Introduction of N_2_ into the discharge plasma is likely to modify this nitridation of the target surface and thus maintain a stable sputter rate during the deposition process.

**Figure 2:**
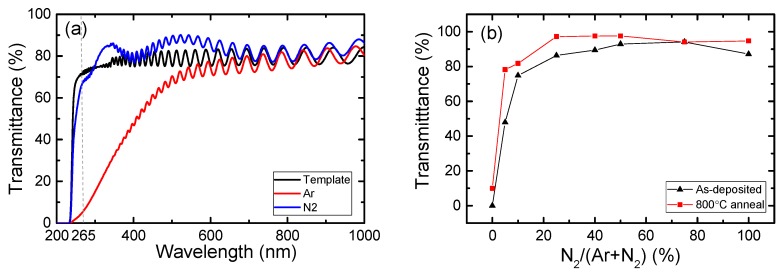
(**a**) Transmission spectra of as-deposited films on AlGaN templates when deposited under Ar or N_2_ plasma atmospheres. (**b**) Optical transmittance at 265 nm for hexagonal boron nitride (h-BN) films vs. various N_2_/(N_2_ + Ar) flow ratios before and after post-annealing treatments at 800 °C.

Figure 2 shows the optical transmittance with changing the mixture ratio of sputtering gases. The optical transmittance spectra of h-BN films deposited under a pure gas ambient (either Ar or N_2_) are shown in Figure 2a. For reference, the transmittance of the template used in this project was also plotted. The results confirm that both samples show excellent transparency in the infrared region (700–1000 nm). However, the sample that was deposited under the pure Ar gas atmosphere shows a long absorption tail from approximately 600 nm to the short wavelength region. In contrast, the transmittance of the h-BN film deposited in the N_2_ gas ambient remains high down to a wavelength of approximately 300 nm. However, the transmittance then decreases very rapidly in the 300−250 nm range. The Al_0.7_Ga_0.3_N template consists of a sapphire substrate, an AlN buffer layer, and an Al_0.7_Ga_0.3_N layer. There are several interfaces after the deposition of h-BN on such a template. The small peak around 340 nm arises from the reduction of Fresnel reflection due to reflection oscillation among the several interfaces [30].

The optical transmittance of h-BN in the DUV region was then explored while varying the gas mixture ratio of N_2_ and Ar. Figure 2b shows the effect of the N_2_ fraction on the transmittance at 265 nm, which is the most effective wavelength for disinfection applications. The transmittance was determined by comparing the transmittance values of the reference template before and after it was coated with the h-BN film. The Fresnel reflections occurring at the interfaces were also taken into account in the calculations. The film thicknesses were calibrated to be equivalent to 200 nm for all samples. The results show that the transmittance reaches a maximum of 94% at a N_2_ percentage of 75%. Initially, the transmittance for the film deposited in the pure Ar gas atmosphere was extremely low (approximately 5%). With increasing introduction of N_2_ gas into the Ar gas, the transmittance increased significantly to 74% at 10% N_2_ content, and then exceeded 94% when the N_2_ percentage increased further to 75%. A pure N_2_ atmosphere can cause a slight reduction in optical transmittance to 87%. In situations where the N_2_ gas is absent or low, the strong absorption in the DUV region is mainly caused by point defects in the form of the nitrogen vacancies (V_N_) that form during deposition [9,31]. The V_N_ vacancies were confirmed by the X-ray photoelectron spectroscopy (XPS) measurement. When N_2_ is introduced into the discharge plasma, the excess nitrogen atoms/ions are believed to play an important role in suppressing V_N_ formation in the deposited films. In addition, the nitrogen molecules (N_2_) may help to form weak bonds, which is beneficial for the synthesis of sp^2^-bonded BN [32]. Although the presence of the N_2_ plasma is beneficial for h-BN film formation, the use of pure N_2_ plasma leads to the film transmittance being much lower than that of films deposited using mixed N_2_ and Ar gases. This occurs because while N-rich conditions allow for V_N_ formation to be suppressed, the transmittance is affected by self-interstitial defects that form easily in the h-BN thin films under N-rich conditions when grown by ion-bombardment-assisted deposition techniques [31]. The post-annealing treatments at 800 °C can increase the optical transmittance of most samples, with the exception of those deposited under the 75% N_2_ atmosphere, which have relatively high optical transmittance before annealing. As a result, the transmittance values of all samples deposited with N_2_ ratios of more than 25% can be improved to exceed 94%. The highest transmittance was 98%, which was recorded for the sample deposited under the 25% N_2_ atmosphere after the annealing treatment. These results indicate that both the nitrogen vacancies and the self-interstitial defects can be repaired somewhat via the high-temperature annealing treatment.

**Figure 3:**
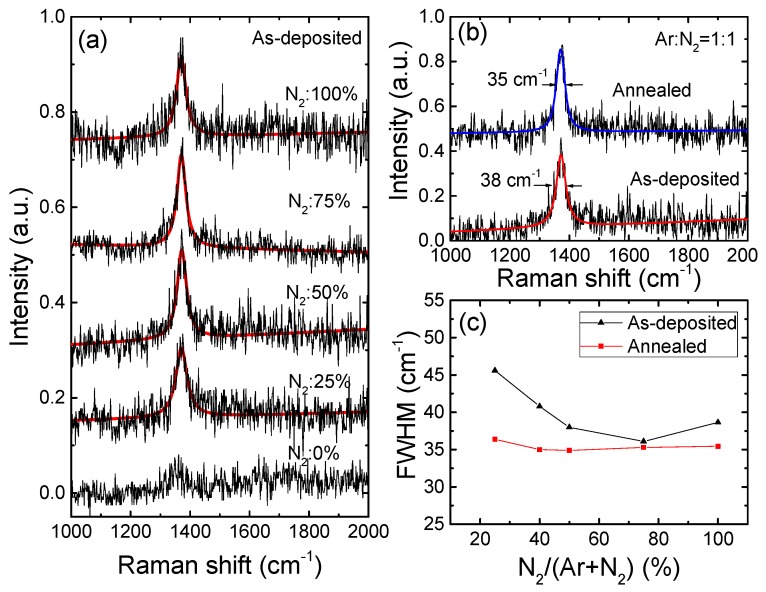
(**a**) Raman spectra of as-deposited h-BN films and Lorentzian fittings for various N_2_/(N_2_ + Ar) gas flow ratios. (**b**) Comparison of Raman spectra for a sample deposited at N_2_:Ar = 1:1 before and after annealing. (**c**) Full width at half maximum (FWHM) of the Raman spectra vs. various N_2_/(N_2_ + Ar) flow ratios before and after post-annealing treatments.

Figure 3 gives the Raman spectra to analyze the phases and crystal quality of BN films. The BN with the hexagonal phase (sp^2^) is favorable because of its p-type doping feasibility, which is a unique property of wide-bandgap materials [9,10,11,12]. Nevertheless, mixed phases composed of sp^2^ and sp^3^ B–N bonds can easily be formed in a BN film, depending on the substrate material and the preparation methods. We next examined the crystalline phase composition of the BN film by Raman scattering spectroscopy. Figure 3a shows the Raman spectra of samples deposited using various N_2_/(Ar + N_2_) gas contents. The peak fittings for the spectra were performed using a Lorentzian function [33]. For the BN film deposited in the pure Ar gas atmosphere, a very broad but weak peak was observed that indicated the poor crystalline quality of the film. When N_2_ gas content of more than 25% was introduced into the discharge plasma, an evident peak was observed for each sample at approximately 1371 cm^−1^, which corresponds to the in-plane E_2g_ phonon mode of sp^2^-bonded BN [8]. If any cubic BN had been mixed into the h-BN film, two other peaks would then have been present at approximately 1056 and 1304 cm^−1^, corresponding to the transverse optical (TO) and longitudinal optical (LO) phonons of the sp^3^ hybridization of the B–N bonds, respectively [34]. The absence of these or any other peaks in the figure indicates that a relatively pure hexagonal-phase BN film was formed. These results can be attributed to the small lattice mismatch that occurs between h-BN and the Al_0.7_Ga_0.3_N template via five-to-four atom alignments at the interface [15]. While annealing at higher temperatures leads to considerably increased optical transmission, it was previously reported that h-BN is unstable at high temperatures and can easily transform into the cubic phase (c-BN) [35]. We also examined the phase compositions of all samples after the annealing treatments in N_2_ at 800 °C via Raman scattering measurements. As an example, Figure 3b shows the Raman spectra for the sample deposited at a N_2_ percentage of 50% before and after annealing. The similar peak positions demonstrate that the h-BN phase does not change after the thermal annealing in our case. The small shift in the peak frequency is most likely to stem from changes in the material strain after the annealing treatment. To identify the crystalline quality of the film, the variation in the full width at half maximum (FWHM) value of the Raman spectra is illustrated in Figure 3c. It was impossible to achieve good fitting for the samples deposited at N_2_ fractions of less than 25%. At the N_2_ percentage of 25%, the FWHM was approximately 45 cm^−1^. The FWHM has a minimum of 36 cm^−1^ at the N_2_ fraction of 75% and this corresponds to the highest optical transmittance shown in Figure 2b. The FWHM values of the peaks were narrowed to approximately 35 cm^−1^ by the thermal annealing treatment for samples deposited under N_2_ atmospheric compositions of more than 40%. The narrowing of the FWHM indicates improvement in the h-BN film crystallinity after the thermal treatment. With reference to Figure 2b and Figure 3c, we can conclude that the enhanced DUV optical transmittance is consistent with better crystalline quality for the h-BN films. The FWHM values are larger than those for single crystal h-BN (9 cm^−1^) or for epi-grown h-BN on sapphire or catalytic metal layers (<20 cm^−1^) [8,24,36], which indicates that the crystalline quality of the h-BN films in this project are still not as good as those obtained at higher growth temperatures.

We would like to point out that the change of the RF plasma power could affect the deposition rate, but it has little influence on the optical transmittance when the RF power was changed in the range from 200 to 70 W.

**Figure 4:**
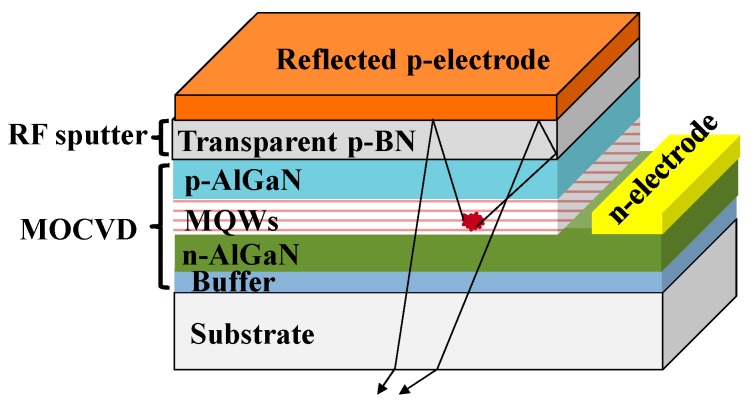
A schematic of a light-emitting device using h-BN film deposited by low-temperature sputtering.

The absorption of the DUV light in the p-side of the present DUV LEDs is one of the major hurdles to realizing highly efficient emitters. In this project, we provide an approach for deposition of highly-DUV–transparent h-BN films on Al_0.7_Ga_0.3_N layers at low temperature. It was previously reported that h-BN can feasibly realize p-type conductivity with a high hole concentration [9] and can also form good ohmic contacts to metal electrodes [23]. In addition, h-BN is an excellent material for both hole injection and electron blocking with respect to Al_0.7_Ga_0.3_N [15]. As a result of the unique characteristics described above, we anticipate that h-BN can be used to replace opaque p-GaN in DUV light-emitting devices in future, as illustrated schematically in Figure 4. A DUV–transparent h-BN layer would act as a multifunctional layer; it is a current spreading layer for current spreading to the entire chip, an effective electron blocking layer that prevents the electron from overflowing to the p-regime, and a good ohmic contact layer to form low contact resistance to metal. Specifically, the high transmittance of h-BN enables the application of various techniques based on multiple reflections to extract light efficiently from the DUV optical devices [37,38,39].

## 4. Conclusions

In conclusion, we have demonstrated deposition of a thick h-BN film by low-temperature RF sputtering on an Al_0.7_Ga_0.3_N layer, which is the material that is commonly used in current sub-280 nm AlGaN-based LEDs. The hexagonal phases of these films were confirmed by Raman spectra measurements. Ar/N_2_ gas mixture ratio optimization during sputtering and the application of a post-annealing treatment can cause the transmittance to reach as high as 98% for 265 nm UVC light in a 200-nm-thick film. In addition, these deposition processes were performed at a low temperature (300 °C). This project thus presents a feasible way to fabricate highly DUV–transparent h-BN films on current sub-280 nm AlGaN-based LEDs at low temperatures.

## References

[1] Inoue S., Naoki T., Kinoshita T., Obata T., Yanagi H. (2015). Light extraction enhancement of 265 nm deep-ultraviolet light-emitting diodes with over 90 mW output power via an AlN hybrid nanostructure. Appl. Phys. Lett..

[2] Inoue S., Tamari N., Taniguchi M. (2017). 150 mW deep-ultraviolet light-emitting diodes with large-area AlN nanophotonic light-extraction structure emitting at 265 nm. Appl. Phys. Lett..

[3] Kolbe T., Knauer A., Rass J., Cho H.K., Hagedorn S., Einfeldt S., Kneissl M., Weyers M. (2017). Effect of Electron Blocking Layer Doping and Composition on the Performance of 310 nm Light Emitting Diodes. Materials.

[4] Khramtsov I.A., Fedyanin D.Y. (2019). Superinjection of Holes in Homojunction Diodes Based on Wide-Bandgap Semiconductors. Materials.

[5] Li L., Zhang Y., Xu S., Bi W., Zhang Z.H., Kuo H.C. (2017). On the Hole Injection for III-Nitride Based Deep Ultraviolet Light-Emitting Diodes. Materials.

[6] Gu Z., Ban S.L., Jiang D.D., Qu Y. (2017). Effects of two-mode transverse optical phonons in bulk wurtzite AlGaN on electronic mobility in AlGaN/GaN quantum wells. J. Appl. Phys..

[7] Watanabe K., Taniguchi T., Kanda H. (2004). Direct-bandgap properties and evidence for ultraviolet lasing of hexagonal boron nitride single crystal. Nat. Mater..

[8] Kubota Y., Watanabe K., Tsuda O., Taniguchi T. (2007). Deep ultraviolet light-emitting hexagonal boron nitride synthesized at atmospheric pressure. Science.

[9] He B., Zhang W.J., Yao Z.Q., Chong Y.M., Yang Y., Ye Q., Pan X.J., Zapien J.A., Bello I., Lee S.T. (2009). P-type conduction in beryllium-implanted hexagonal boron nitride films. Appl. Phys. Lett..

[10] Nose K., Oba H., Yoshida T. (2006). Electric conductivity of boron nitride thin films enhanced by in situ doping of zinc. Appl. Phys. Lett..

[11] Dahal R., Li J., Majety S., Pantha B.N., Cao X.K., Lin J.Y., Jiang H.X. (2011). Epitaxially grown semiconducting hexagonal boron nitride as a deep ultraviolet photonic material. Appl. Phys. Lett..

[12] Lu M., Bousetta A., Bensaoula A., Waters K., Schultz J.A. (1996). Electrical properties of boron nitride thin films grown by neutralized nitrogen ion assisted vapor deposition. Appl. Phys. Lett..

[13] Attaccalite C., Bockstedte M., Marini A., Rubio A., Wirtz L. (2011). Coupling of excitons and defect states in boron-nitride nanostructures. Phys. Rev. B.

[14] Laleyan D.A., Zhao S., Woo S.Y., Tran H.N., Le H.B., Szkopek T., Guo H., Botton G.A., Mi Z. (2017). AlN/h-BN Heterostructures for Mg Dopant-Free Deep Ultraviolet Photonics. Nano Lett..

[15] Hao G.D., Tsuzuki S., Inoue S. (2019). Small valence band offset of h-BN/Al0.7Ga0.3N heterojunction measured by X-ray photoelectron spectroscopy. Appl. Phys. Lett..

[16] Sun F., Hao Z., Liu G., Wu C., Lu S., Huang S., Liu C., Hong Q., Chen X., Cai D. (2018). P-Type conductivity of hexagonal boron nitride as a dielectrically tunable monolayer: Modulation doping with magnesium. Nanoscale.

[17] Cho H., Park S., Won D.I., Kang S.O., Pyo S.S., Kim D.I., Kim S.M., Kim H.C., Kim M.J. (2015). Growth kinetics of white graphene (h-BN) on a planarised Ni foil surface. Sci. Rep..

[18] Uchida Y., Iwaizako T., Mizuno S., Tsuji M., Ago H. (2017). Epitaxial chemical vapour deposition growth of monolayer hexagonal boron nitride on a Cu(111)/sapphire substrate. Phys. Chem. Chem. Phys..

[19] Kim S.M., Hsu A., Park M.H., Chae S.H., Yun S.J., Lee J.S., Cho D.H., Fang W., Lee C., Palacios T. (2015). Synthesis of large-area multilayer hexagonal boron nitride for high material performance. Nat. Commun..

[20] Kobayashi Y., Akasaka T. (2008). Hexagonal BN epitaxial growth on (0001) sapphire substrate by MOVPE. J. Cryst. Growth.

[21] Snure M., Paduano Q., Hamilton M., Shoaf J., Mann J.M. (2014). Optical characterization of nanocrystalline boron nitride thin films grown by atomic layer deposition. Thin Solid Films.

[22] Hu C., Kotake S., Suzuki Y., Senoo M. (2000). Boron nitride thin films synthesized by reactive sputtering. Vacuum.

[23] Majety S., Li J., Cao X.K., Dahal R., Pantha B.N., Lin J.Y., Jiang H.X. (2012). Epitaxial growth and demonstration of hexagonal BN/AlGaN p-n junctions for deep ultraviolet photonics. Appl. Phys. Lett..

[24] Ohta J., Fujioka H. (2017). Sputter synthesis of wafer-scale hexagonal boron nitride films via interface segregation. APL Mater..

[25] Chapman B. (1980). Glow Discharge Processes: Sputtering and Plasma Etching.

[26] Deng J.X., Zhang X.K., Yao Q., Wang X.Y., Chen G.H., He D.Y. (2009). Optical properties of hexagonal boron nitride thin films deposited by radio frequency bias magnetron sputtering. Chin. Phys. B.

[27] Lee J.-S., Byun D., Oh H.-K., Choi Y.J., Lee H.-Y., Kim J.-H., Lim T.-Y., Hwang J. (2012). Effect of the growth temperature on the properties of AlxGal−xN epilayers grown by HVPE. J. Cryst. Growth.

[28] Kinoshita T., Hironaka K., Obata T., Nagashima T., Dalmau R., Schlesser R., Moody B., Xie J., Inoue S., Kumagai Y. (2012). Deep-Ultraviolet Light-Emitting Diodes Fabricated on AlN Substrates Prepared by Hydride Vapor Phase Epitaxy. Appl. Phys. Express.

[29] Todi V.O., Shantheyanda B.P., Todi R.M., Sundaram K.B., Coffey K. (2011). Optical characterization of BCN films deposited at various N_2_/Ar gas flow ratios by RF magnetron sputtering. Mater. Sci. Eng. B.

[30] Mouchart J. (1977). Thin film optical coatings. 1. Optical coating stabilities. Appl. Opt..

[31] Orellana W., Chacham H. (2001). Stability of native defects in hexagonal and cubic boron nitride. Phys. Rev. B.

[32] Mieno M., Yoshida T. (1990). Preparation of Cubic Boron-Nitride Films by RF-Sputtering. Jpn. J. Appl. Phys..

[33] Ferrari A.C., Robertson J. (2000). Interpretation of Raman spectra of disordered and amorphous carbon. Phys. Rev. B.

[34] Zhang W.J., Chong Y.M., Bello I., Lee S.T. (2007). Nucleation, growth and characterization of cubic boron nitride (cBN) films. J. Phys. D Appl. Phys..

[35] Zhang X.K., Deng J.X., Wang L., Wang X.Y., Yao Q., Chen G.H., He D.Y. (2008). Phase transformation in BN films by nitrogen-protected annealing at atmospheric pressure. Appl. Surf. Sci..

[36] Jiang H.X., Lin J.Y. (2014). Hexagonal boron nitride for deep ultraviolet photonic devices. Semicond. Sci. Technol..

[37] Hao G.D., Wang X.L. (2013). Enhancement of light-extraction efficiency in AlGaInP light-emitting diodes using evanescent wave coupling effect. Appl. Phys. Lett..

[38] Taniguchi M., Hao G., Nakaya K., Inoue S. (2015). Optimization of AlN Substrate Geometry for AlGaN-Based Deep-Ultraviolet Light-Emitting Diodes. Proceedings of the 2015 11th Conference on Lasers and Electro-Optics Pacific Rim (CLEO-PR).

[39] Zhao J., Ding X., Miao J., Hu J., Wan H., Zhou S. (2019). Improvement in Light Output of Ultraviolet Light-Emitting Diodes with Patterned Double-Layer ITO by Laser Direct Writing. Nanomaterials.

